# Comparative Analysis of Markerless Motion-Capture Models for Assessing Football Kinematics During 30 m Long-Pass Tasks

**DOI:** 10.3390/s26123654

**Published:** 2026-06-08

**Authors:** Donghao Wang, Junkai Yu, Shiqin Chen, Jingran Yang, Weichao Jiang, Yikang Gong, Chong Luo

**Affiliations:** 1 School of Physical Education, Shanghai University of Sport, Shanghai 200438, China; 24300504@sus.edu.cn (D.W.); 2421111031@sus.edu.cn (Y.G.); 2 School of Physical Education, Huzhou University, Huzhou 313000, China; 202211320131@stu.zjsru.edu.cn; 3 School of Athletic Sport, Shanghai University of Sport, Shanghai 200438, China; chenshiqin0507@163.com (S.C.); 2421852008@sus.edu.cn (W.J.); 4 School of Physical Education and Health, East China Normal University, Shanghai 200241, China; 10231310055@stu.ecnu.edu.cn

**Keywords:** football, 30 m long pass, markerless motion capture, kinematics, MediaPipe Pose, DWPose

## Abstract

This study was based on a 30 m inside-foot long-pass scenario and aimed to preliminarily evaluate the agreement between MediaPipe Pose, DWPose, YOLO-Pose, and Xsens, as well as their practical utility under real-field conditions. Twelve elite male football players performed 15 consecutive long-passes, with data collected simultaneously using Xsens and two smartphones positioned at 15° and 35° to the right front of the participants. The Intraclass Correlation Coefficient (ICC (2,1)) and Bland–Altman analysis were used to evaluate discrete kinematic measures. Continuous kinematic agreement was assessed using Root Mean Square Error (RMSE) and the Coefficient of Multiple Determination (CMD), while Statistical Parametric Mapping (SPM) and Statistical non-Parametric Mapping (SnPM) compared differences across the entire analysis interval. Across the three models, CMD ranged from 0.13 ± 0.17 to 0.67 ± 0.25, and RMSE ranged from 9.88 ± 8.20° to 39.92 ± 10.44°. The SPM and SnPM results showed that significant differences were mainly concentrated in the bilateral hip, knee, and ankle joints. The three models cannot yet be used for field-based high-precision kinematic data measurement; however, MediaPipe Pose and DWPose may be selectively used for rapid screening of movement patterns and analysis of movement trends in football-specific technical movements.

## 1. Introduction

Football is one of the most popular sports worldwide and has long attracted research attention because of its broad participation base and highly competitive nature. In football-specific research, accurately obtaining kinematic information on players’ technical movements is important for elucidating movement execution mechanisms, evaluating technical performance, and optimizing training interventions. With the continued development of sports biomechanics, motion capture has become an important tool for the quantitative analysis of football-specific technical movements. Using motion-capture technology, researchers can obtain kinematic data during the execution of technical movements in a relatively objective manner, thereby identifying movement characteristics, comparing movement differences, evaluating movement performance, and providing scientific evidence for technical optimization, movement correction, and injury prevention in players [1,2].

Among existing motion-capture technologies, marker-based optical motion-capture systems are widely used in movement analysis research because of their high measurement accuracy [3]. They are also commonly regarded as reference standards for evaluating the reliability and validity of markerless motion-capture systems and inertial motion-capture systems under laboratory conditions [4,5]. However, optical systems typically rely on fixed laboratory spaces, complex marker placement, and multi-camera calibration, making it difficult to collect complete kinematic data over large areas and under natural conditions in open environments such as football fields. In comparison, inertial motion-capture systems have clear advantages in field adaptability and portability. Such systems represented by Xsens attach multiple inertial measurement units (IMUs) to the human body and combine sensor-fusion algorithms with biomechanical models to achieve real-time three-dimensional capture of human movements [6]. Previous studies have shown that Xsens demonstrates higher agreement with optical motion-capture systems in the measurement of joint angles and key kinematic parameters during sport-specific technical movements, daily movements, jumping, and change-of-direction tasks [4,7,8,9]. In football-related contexts, Xsens has shown only small errors compared with optical systems in measuring knee joint angles of the support and kicking legs [9]. Moreover, Xsens has been used to measure joint kinematics during a range of rapid dynamic movements in football research [10,11,12], indicating that it has relatively high reference value in complex movement contexts outside the laboratory. Therefore, the present study used Xsens as a field-deployable reference system for evaluating the performance of markerless motion-capture models, rather than as an absolute error-free gold standard.

Although inertial motion-capture systems have strong portability and field adaptability, their measurements may still be affected by data drift, magnetic interference, and soft tissue artifacts [13,14], which may limit their measurement stability during highly dynamic and complex sport-specific movements. In recent years, with the rapid development of computer vision and deep learning, markerless motion capture has gradually become an important direction for kinematic assessment because it does not require marker placement, imposes fewer restrictions on the testing environment, and can adapt to complex movement scenarios, multi-person interaction scenarios, and natural movement conditions [13]. At present, most 2D markerless motion-capture models are constructed based on convolutional neural networks (CNNs) or related deep-learning frameworks [15]. On this basis, multi-camera acquisition can further enable three-dimensional reconstruction; for example, OpenCap has achieved accuracy close to that of IMU systems and commercial markerless systems in some tasks [16]. Previous studies have validated the accuracy of several 2D markerless motion-capture models in relatively standardized tasks such as gait [17,18,19,20,21]. In football, a study based on an OpenPose-reconstructed three-dimensional markerless motion-capture model obtained kinematic data with relatively high accuracy in an unopposed shooting context [22].

However, although three-dimensional markerless motion capture can theoretically provide more complete spatial movement information, its application in open environments such as football fields often depends on multi-camera synchronization, spatial calibration, and relatively stable filming conditions. These requirements increase implementation cost and time and are not conducive to the rapid assessment and timely feedback of football-specific technical movements. In contrast, mobile-device-based 2D markerless motion capture has certain limitations in the completeness of spatial information, but it has clear advantages in ease of setup, cost, and feedback efficiency, making it more suitable for a field-based rapid assessment of football-specific movements. Joint angles estimated by 2D motion-capture models from single-view 2D images are essentially projections of three-dimensional limb-segment movement onto the image plane; therefore, these estimates may differ from true three-dimensional joint angles [23]. Football-specific technical movements usually involve high-speed swinging and rotation of multiple body segments in three-dimensional space. When the joint movement plane deviates substantially from the image plane, true three-dimensional angles may be compressed or distorted during projection and may be further affected by limb occlusion. It follows that the camera viewing angle, together with the degree of correspondence between the image plane and the joint’s primary plane of motion, is an important factor influencing the accuracy of 2D joint-angle measurements. At present, MediaPipe Pose has been validated against reference standards in gait experiments [21], and YOLO-Pose and DWPose are increasingly being applied in sports movement analysis. However, direct evidence regarding the measurement accuracy and applicability of these three models in football-specific technical movements remains limited.

In real matches, the 30 m inside-foot long-pass is an important technical component of football [24]. It plays an important role in vertical progression, rapid penetration of the opposing defensive line, and the creation of attacking space, and therefore has high tactical value [25]. This technical movement is also closely related to the attacking performance of teams and players. From a movement perspective, it is characterized by typical proximal-to-distal sequencing [26]. Its execution relies on coordinated movement of the trunk, pelvis, and lower-limb segments to effectively amplify distal-segment velocity at ball contact [27].

In summary, the present study simulated an unopposed 30 m inside-foot long-pass scenario in real match contexts. The objective of this study was to preliminarily evaluate the agreement of three 2D markerless motion-capture models, namely MediaPipe Pose, DWPose, and YOLO-Pose, when used with mobile devices, against a field-deployable inertial motion-capture reference system. A further objective was to assess their feasibility for the rapid screening of movement patterns and analysis of movement trends in football-specific technical movements. Given the inherent limitations of using 2D projection to quantify three-dimensional multiplanar movement, this study hypothesized that the agreement between the three markerless motion-capture models and the field-deployable reference system would show joint specificity and camera-view dependence. For joints whose movement planes remained closer to the image plane throughout the movement cycle, relatively higher agreement was expected; conversely, greater mismatch between the movement plane and the image plane was expected to increase quantification differences.

## 2. Materials and Methods

### 2.1. Study Design

This study adopted a cross-sectional method-comparison design. Xsens and 2D video data were collected under outdoor football-field conditions, and the same 30 m inside-foot long-pass movement was quantified using MediaPipe Pose, DWPose, and YOLO-Pose. With Xsens used as a field-deployable reference system, the agreement of the three 2D markerless motion-capture models in kinematic measures was compared.

### 2.2. Participants

Twelve elite male football players from Shanghai University of Sport participated in this study (age: 20.4 ± 1.2 years; height: 175.0 ± 5.2 cm; mass: 72.0 ± 7.2 kg). The inclusion criteria were membership of the Shanghai University of Sport football team, a self-reported right-preferred kicking leg, and no injury within the previous six months. Before testing, all participants were informed of this study’s purpose, experimental procedures, potential risks, and intended use of the data, and they voluntarily provided written informed consent. During this study, all participant information and experimental data were managed according to a confidentiality protocol. The data were used only for the analyses in this study, and access was restricted to the research team. This study was approved by the Scientific Research Ethics Committee of Shanghai University of Sport, with the approval number 102772025RT241.

### 2.3. Instruments and System Setup

#### 2.3.1. Xsens Inertial Motion-Capture System

The Xsens motion-capture system (Enschede, The Netherlands) was used as the field-deployable reference system. Seventeen inertial measurement units were used for full-body motion capture. Before testing, the system was calibrated according to the manufacturer’s recommended procedures, including an N-pose static calibration and a short-distance back-and-forth walking dynamic calibration. The sampling frequency was set at 240 Hz, and the *X*-axis of the global coordinate system pointed toward the participant’s forward direction. Joint definitions followed the recommendations of the International Society of Biomechanics (ISB) [28,29,30], and joint-angle outputs were based on the intermediate local coordinate systems used by the Xsens system.

#### 2.3.2. Video Acquisition System

Two iOS smartphones were used for 2D video acquisition: an iPhone 15 Pro and an iPhone 16 Pro Max (Apple Inc., Cupertino, CA, USA). Based on the results of pilot testing, positioning the cameras at 15° and 35° to the right front of the participant helped maintain the positional identification accuracy of trunk and lower-limb keypoints during the long-pass task and reduced occlusion between limbs to some extent. Therefore, these two camera views were selected as the video acquisition positions under practical field conditions. The iPhone 15 Pro was positioned at 15°, and the iPhone 16 Pro Max was positioned at 35°. The two camera views were not intended for three-dimensional reconstruction. Instead, they were used to compare the ability of 2D markerless motion-capture models to quantify the kinematic trends of the long-pass movement under practically feasible field-filming conditions. The camera angle was defined as the angle between the line connecting the recording device to the ball-placement origin and the initial test line. Both devices were positioned 3 m from the long-pass starting point, with the lens height set at 0.80 m. Videos were recorded using the main camera at 1× focal length, with a resolution of 1920 × 1080 pixels and a frame rate of 60 Hz.

#### 2.3.3. Two-Dimensional Markerless Motion-Capture Models

**Figure 1:**
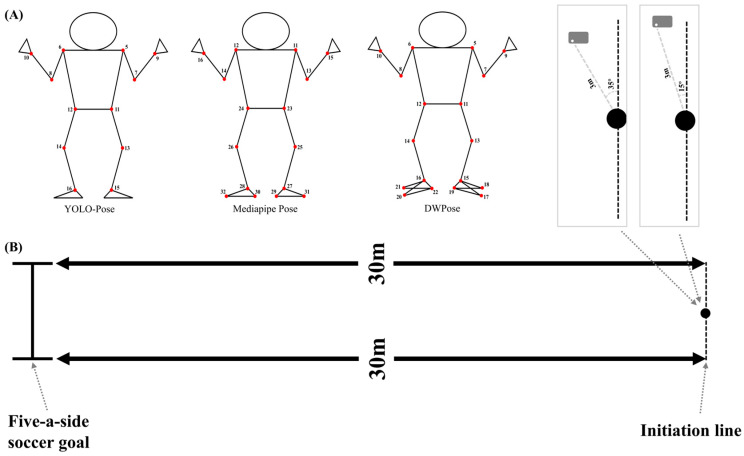
Main joint keypoints identifiable by the three 2D markerless motion-capture models and the experimental field setup. Note: (**A**) Main body keypoints identifiable by YOLO-Pose, MediaPipe Pose, and DWPose, and included in joint-angle calculation. (**B**) Schematic illustration of the experimental field setup for the 30 m inside-foot long-pass test. The distance between the starting line and the five-a-side goal was 30 m; two smartphones were positioned at 15° and 35° to the right front of the participant.

Three 2D markerless motion-capture models were selected for comparison: (1) YOLO-Pose, using the YOLOv11x-pose pretrained model trained on the COCO-Pose dataset; (2) DWPose, using the DWPose-l_384 × 288 pretrained model, which was developed based on the MMPose framework and trained with public datasets including COCO-WholeBody [31]; and (3) MediaPipe Pose, using the pose_landmarker_heavy pretrained model developed based on the BlazePose framework [32]. All three models performed inference using publicly released pretrained weights. The minimum confidence threshold for keypoint detection was uniformly set at 0.30 to ensure comparable output conditions across models. The three markerless motion-capture models differed in the number of identifiable body keypoints. Excluding detailed facial and hand keypoints, the main body keypoints used for joint-angle calculation in this study are shown in Figure 1A. The red points indicate the keypoints that could be identified by each model and were included in the analysis. 

### 2.4. Experimental Procedures

Testing was conducted on a standard football field. After completing a standardized warm-up, all participants wore the Xsens system and sequentially performed the 30 m inside-foot long-pass test. Participants were required to complete 15 consecutive long passes using the right foot. In all trials, the right leg was used as the kicking leg and the left leg as the support leg; therefore, the left knee represented the support-leg knee joint, and the right knee represented the kicking-leg knee joint. A five-a-side goal was used as the target, and the straight-line distance from the long-pass starting line to the goal line was 30 m. The rest interval between long-passes was in the range of 30–60 s. All trials were included regardless of whether the ball entered the goal. In each trial, the Xsens system and the two mobile recording devices simultaneously recorded movement data. All videos covered the complete approach, support, swing, ball contact, and follow-through processes to ensure the completeness of subsequent movement-sequence segmentation and key joint-kinematics analysis.

### 2.5. Data Processing and Outcome Measures

#### 2.5.1. Movement-Cycle Segmentation

Xsens raw data were collected at a sampling frequency of 240 Hz and were exported using the official software’s 4:1 downsampling option. Some kinematic features may not have been fully retained during the downsampling process. Therefore, the comparison results of this study mainly reflect the ability of 2D markerless motion-capture models to estimate movement trends throughout the long-pass process under 60 Hz video acquisition conditions. This study used a temporal alignment method based on predefined kinematic events. The analysis window was segmented using the right knee joint as the reference joint. The movement onset was defined as the first time point at which the right knee joint angle began to decrease continuously. This onset point corresponded to the stage at which the kicking leg began the backswing and the support leg gradually approached the support location. Subsequently, 30 consecutive frames were extracted from this onset point for comparative analysis between Xsens and the 2D markerless motion-capture models. The discrete indicators were divided into the backswing phase and the follow-through phase. During the backswing phase, the left knee joint angle first increased and then decreased, and the turning point was used as the comparison time point for the backswing phase. Throughout the follow-through phase, the right knee joint angle first increased and then decreased, and this turning point was used as the comparison time point for the follow-through phase.

#### 2.5.2. Data Processing

For each frame, joint angles based on the ZXY Euler sequence were synthesized into an absolute angle to represent the angle between adjacent segments. The calculation rules were as follows: (1) hip joint absolute angle = 180° − segment angle; (2) shoulder joint absolute angle = segment angle; (3) under dorsiflexion, ankle joint angle = 90° − segment angle, whereas under plantarflexion, ankle joint angle = 90° + segment angle; and (4) for the knee and elbow joints, because of their joint movement characteristics, the flexion–extension angles of the knee and elbow were approximated as the absolute angles of these joints for comparative calculations. When occlusion between limbs caused missing data in the markerless motion-capture outputs, the missing values were filled using a Piecewise Cubic Hermite Interpolating Polynomial, and boundary missing values were held constant. Trials with more than three filled values were discarded. The raw data were then filtered using a Savitzky–Golay filter [33], with a window length of 5 and a second-order polynomial.

### 2.6. Statistical Analysis

Data analyses were performed using Python 3.10 and 3.11. The Intraclass Correlation Coefficient, ICC (2,1), was used to assess the agreement between the three markerless motion-capture models and Xsens [34]. ICC values of <0.50, 0.50–0.75, 0.75–0.90, and >0.90 were interpreted as poor, moderate, good, and excellent agreement, respectively [35]. Bland–Altman analysis was also used to evaluate systematic bias (Bias) and the 95% limits of agreement (95% LoA) between the two measurement methods. Statistical analyses of the movement cycle were divided into two categories: (1) continuous kinematic variables calculated at the individual-trial level; and (2) statistical analyses used for result interpretation after calculating participant-level mean values. For the first category, the Root Mean Square Error (RMSE) was calculated for each movement trial, and the mean and standard deviation across all trials were obtained. An RMSE ≤ 5° indicated excellent agreement, whereas an RMSE > 5° indicated poor agreement [4,7,35]. The Coefficient of Multiple Determination (CMD) was also used to evaluate joint-angle waveform similarity. Because the waveform comparison in this study involved only a single independent variable and a single dependent variable, R^2^ was used to represent waveform similarity and was graded according to the interpretive framework for CMD used in previous studies: 0–0.50, poor; 0.50–0.74, moderate; 0.75–0.90, good; and 0.90–1.00, excellent [35,36]. For the second category, the frame-by-frame mean angle of each joint was first calculated at the participant level. The spm1d package was then used to perform paired-sample *t*-tests based on Statistical Parametric Mapping (SPM) across the entire movement cycle for each participant [37]. Statistical non-Parametric Mapping (SnPM) based on permutation testing was used for robustness verification [38,39,40]. By combining the SPM and SnPM results, significant differences between the three markerless motion-capture models and Xsens across the entire fixed analysis window were evaluated. The significance level for all tests was set at α = 0.05.

## 3. Results

### 3.1. Discrete Kinematic Measures

The agreement analysis of discrete kinematic variables showed that most results quantified by the three markerless motion-capture models differed significantly from Xsens (Table 1 and Table 2 and Supplementary Materials). Overall, however, the knee joint showed relatively higher agreement. Among the joint angles quantified by MediaPipe Pose, the left knee showed the best agreement. The ICC values were 0.83 and 0.79 in the backswing phase and 0.75 and 0.48 in the follow-through phase. The systematic bias was small (all *p* < 0.05), the agreement remained at a moderate level or above, and the 95% LoA intervals were relatively narrow. For DWPose, the ICC values for the left knee were 0.81 and 0.78, and 0.68 and 0.50, respectively, while the Bias and 95% LoA were slightly poorer than those of MediaPipe Pose. In contrast, agreement for the ankle joint was generally poor. The ICC values of MediaPipe Pose and DWPose were close to 0 and included negative values, accompanied by larger bias (all *p* < 0.05) and wider 95% LoA.

In addition, the three models showed different characteristics in their quantified results. MediaPipe Pose and DWPose generally performed better than YOLO-Pose. The left elbow quantified by MediaPipe Pose showed relatively higher agreement during the backswing phase. Under Camera View 2, the left shoulder quantified by DWPose reached excellent agreement, whereas YOLO-Pose achieved moderate or higher agreement only for a small number of joints, including the left knee and left shoulder (see Supplementary Materials). Bland–Altman analysis showed that the Bias and 95% LoA of most joints still indicated relatively large systematic errors and inter-individual dispersion (Table 1 and Table 2 and Supplementary Materials), particularly for the ankle and upper-limb joints. This suggested that, for these joint angles, the single-measurement results differed substantially between the three markerless motion-capture models and the field-deployable reference system.

### 3.2. Continuous Kinematic Measures

**Figure 2:**
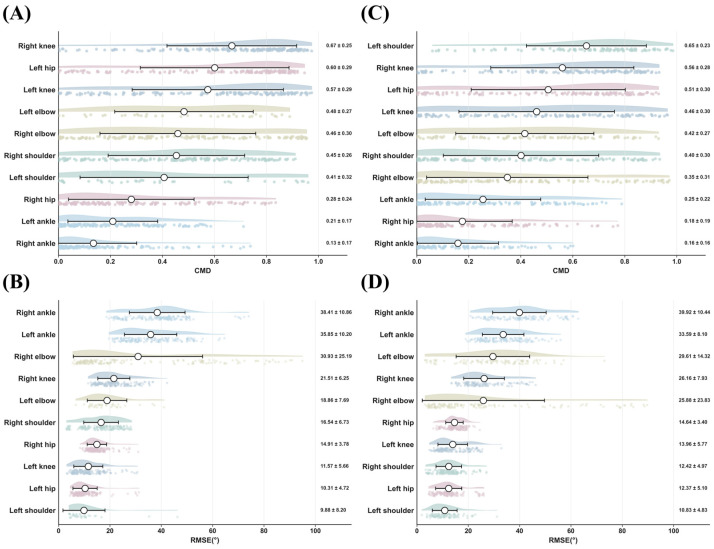
Comparison of joint kinematic agreement between MediaPipe Pose and Xsens at two camera viewing angles (15° and 35°). Note: (**A**,**C**) CMD values representing waveform similarity. (**B**,**D**) RMSE values representing absolute angular differences. Panels (**A**,**B**) correspond to 15°, and (**C**,**D**) correspond to 35°. Data are presented as individual trials with mean ± SD.

**Figure 3:**
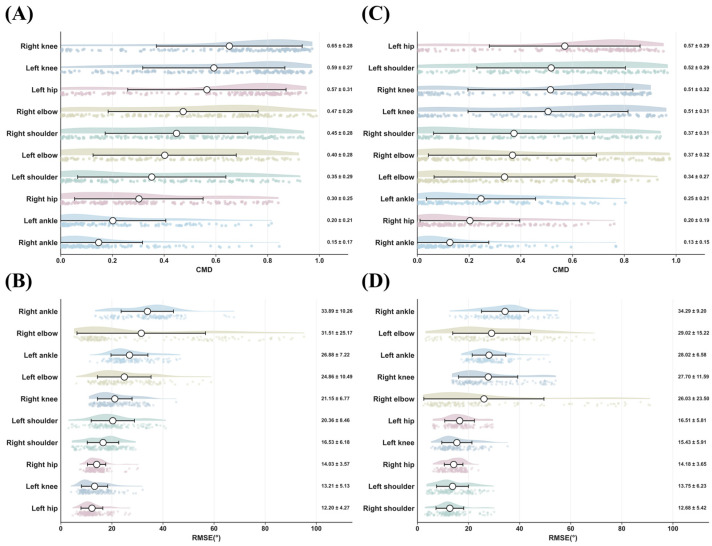
Comparison of joint kinematic agreement between DWPose and Xsens at two camera viewing angles (15° and 35°). Note: (**A**,**C**) CMD values representing waveform similarity. (**B**,**D**) RMSE values representing absolute angular differences. Panels (**A**,**B**) correspond to 15°, and (**C**,**D**) correspond to 35°. Data are presented as individual trials with mean ± SD.

Overall, the quantified results of the continuous kinematic measures obtained from the three markerless motion-capture models showed clear joint specificity and camera-view dependence (Figure 2 and Figure 3 and Supplementary Materials). CMD ranged from 0.13 ± 0.17 to 0.67 ± 0.25, which did not reach the good evaluation range. RMSE values were generally high, ranging from 9.88 ± 8.20 to 39.92 ± 10.44, indicating poor agreement and suggesting that the three motion-capture models have limited interchangeability with Xsens. For DWPose and MediaPipe Pose, the overall performance at the 15° camera view was better than that at the 35° camera view, especially for the bilateral knee joints. CMD values were relatively higher for the right knee, at 0.65 ± 0.28 and 0.67 ± 0.25, and for the left knee, at 0.59 ± 0.27 and 0.57 ± 0.29. This indicated that the measured joint movement trends were relatively similar to those obtained from Xsens across the entire fixed analysis window.

However, the dispersion of the quantified bilateral knee results at the 15° camera view should not be ignored, as reflected by the wide distribution of results across trials. Meanwhile, RMSE remained high. The RMSE values of the right knee for DWPose and MediaPipe Pose were 21.15 ± 6.77 and 21.51 ± 6.25, respectively, and those of the left knee were 13.21 ± 5.13 and 11.57 ± 5.66, respectively. Thus, even when the joints showed better waveform similarity, the absolute angular deviation remained non-negligible. Notably, under the 35° camera view, the waveform similarity of the left shoulder quantified by MediaPipe Pose increased from 0.41 ± 0.32 at the 15° camera view to 0.65 ± 0.23. In addition, both markerless motion-capture models clearly showed low CMD and high RMSE for the bilateral ankle joints (Figure 2 and Figure 3), together with relatively narrow distributions of the quantified results, indicating that the ankle-joint measurement error was not caused by outliers. Considering the results across different joints and camera views, MediaPipe Pose showed a relatively better level of agreement, DWPose showed more balanced performance, and YOLO-Pose generally performed slightly worse than the other two models.

**Figure 4:**
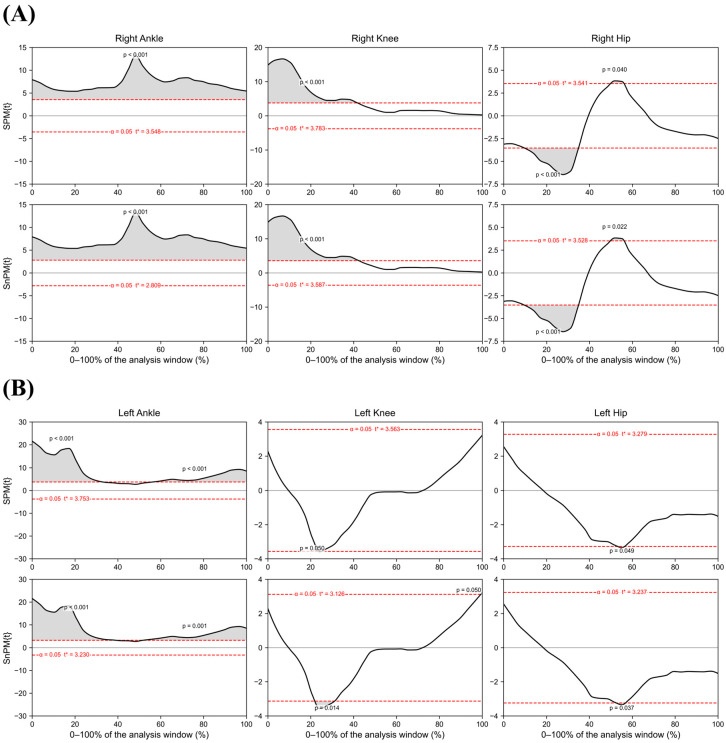
SPM and SnPM comparisons of bilateral lower-limb joint-angle time series between MediaPipe Pose and Xsens over the fixed analysis window (0–100%) at a 15° camera angle. Note: (**A**) Right-side joints, including the right ankle, right knee, and right hip. (**B**) Left-side joints, including the left ankle, left knee, and left hip. Each column represents one joint. For each joint, the upper row presents the SPM{t} result, and the lower row presents the SnPM{t} result. The black solid curve represents the test statistic across the fixed analysis window. Red dashed lines indicate the critical thresholds of the test statistic at α = 0.05, denoted as t* in the figure. Gray shaded regions represent supra-threshold clusters, indicating time regions with significant differences between MediaPipe Pose and Xsens, with corresponding *p* values annotated in the figure. The *x*-axis represents the fixed analysis window (%).

**Figure 5:**
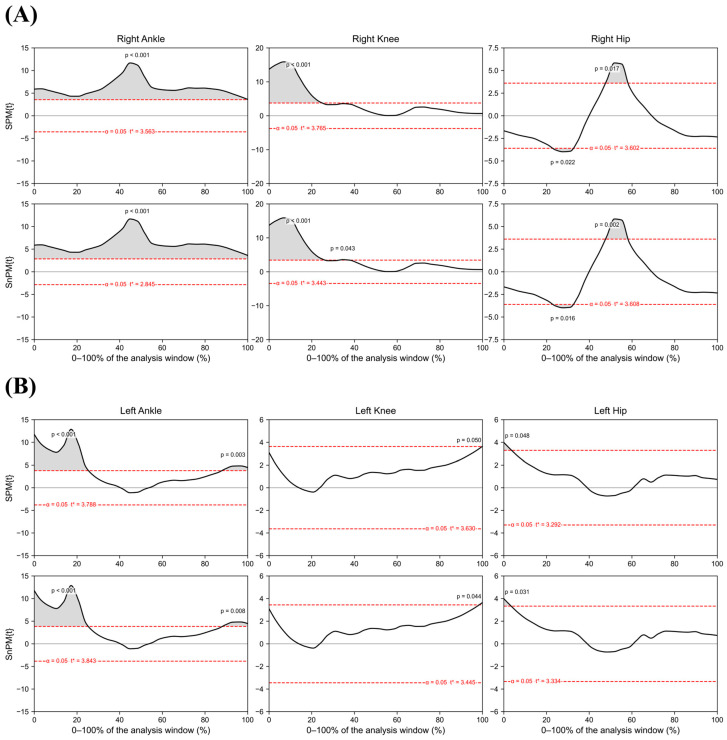
SPM and SnPM comparisons of bilateral lower-limb joint-angle time series between DWPose and Xsens over the fixed analysis window (0–100%) at a 15° camera angle. Note: (**A**) Right-side joints, including the right ankle, right knee, and right hip. (**B**) Left-side joints, including the left ankle, left knee, and left hip. Each column represents one joint. For each joint, the upper row presents the SPM{t} result, and the lower row presents the SnPM{t} result. The black solid curve represents the test statistic across the fixed analysis window.Red dashed lines indicate the critical thresholds of the test statistic at α = 0.05, denoted as t* in the figure. Gray shaded regions represent supra-threshold clusters, indicating time regions with significant differences between DWPose and Xsens, with corresponding *p* values annotated in the figure. The *x*-axis represents the fixed analysis window (%).

The SPM and SnPM results showed that systematic significant differences were mainly concentrated in the bilateral hip, knee, and ankle joints (Figure 4 and Figure 5), whereas significant intervals for the bilateral shoulder and elbow joints were relatively limited. Most of these intervals were confirmed by both SPM and SnPM, with broader regions of difference occurring more often at 35° than at 15°. At 15°, the right ankle angles quantified by DWPose and MediaPipe Pose differed significantly from the Xsens measurements throughout the entire fixed analysis window (0–100%; all *p* < 0.001). Under both camera views, DWPose and MediaPipe Pose showed similar results for the left ankle, with significant difference intervals occurring in the early and late phases of the movement cycle (all *p* < 0.05). The results for the bilateral ankle joints indicated that the quantified ankle angles obtained by the two markerless models showed substantial deviations from Xsens across almost the entire movement window.

In addition, the significant difference intervals of the right knee showed relatively similar temporal distribution characteristics across the different markerless motion-capture models, mainly occurring in the early and middle phases of the movement. In particular, under the 15° camera view, MediaPipe Pose showed significant differences during the early-to-middle regions in the range of 0.0–41.4% (SPM and SnPM: *p* < 0.001). The mean-angle comparison plots also showed clear deviations between MediaPipe Pose, DWPose, and Xsens within the significant regions. Furthermore, differences in the right hip also showed certain temporal distribution characteristics, with difference intervals commonly appearing in the middle phase of the long-pass movement. The mean comparison plots indicated that the markerless motion-capture systems and the field-deployable reference system described two relatively different movement trends. For the left hip, significant difference intervals were affected by changes in camera view. When the camera view changed, although the mean waveforms were relatively similar, a certain overall deviation may have existed. The bilateral shoulder and elbow joints generally showed fewer significant difference intervals, but the mean waveforms showed relatively wide variability.

## 4. Discussion

This study is the first to compare the agreement of three markerless motion-capture models, namely MediaPipe Pose, DWPose, and YOLO-Pose, with Xsens in quantifying the 30 m inside-foot long-pass movement in football. Overall, none of the tested models showed consistently high agreement with Xsens for either discrete or continuous kinematic measures. The mean CMD values ranged approximately from 0.13 to 0.67 and, overall, did not reach the good range. At the current stage, 2D markerless motion capture combined with mobile devices remains difficult to use as a replacement for a field-deployable reference system for high-precision quantification of joint kinematics in complex football-specific movements. Nevertheless, MediaPipe Pose and DWPose still showed a certain ability to identify movement trends for some joints and under specific camera views.

### 4.1. Integrated Interpretation of Discrete and Continuous Measures

The results showed differences between the findings reflected by discrete kinematic measures and continuous kinematic measures. Some joints showed moderate or good agreement in discrete kinematic measures. For example, the left shoulder joint quantified by DWPose during the follow-through phase showed ICC values of 0.66 and 0.80 under the two camera views (Table 2), and the left elbow joint quantified by MediaPipe Pose during the backswing phase showed ICC values of 0.66 and 0.76 (Table 1). However, these joints showed limited waveform similarity and generally high RMSE in the continuous measures. Under the 15° camera view, the right knee joint quantified by MediaPipe Pose and DWPose during the backswing phase showed ICC values of 0.10 and 0.13, respectively. In contrast, this joint showed the best overall performance in the continuous measures, with relatively high waveform similarity and lower RMSE.

This discrepancy may mainly be explained by differences in the input data used for calculating the two types of measures. Discrete kinematic measures focus on angle agreement at key time points during the movement and are more likely to capture the stability of angle quantification in short-cycle movements. Continuous kinematic measures are more closely related to the overall differences across the long-pass movement. For technical movements such as the 30 m long pass, which involve rapid joint movement and high explosiveness, slight time-series alignment errors and keypoint jitter are more likely to occur. In comparison, continuous kinematic measures have stronger resistance to such interference and may better reflect the ability of markerless motion-capture models to reconstruct movement trends during the long-pass task.

### 4.2. Influence of Movement Plane Changes

Camera view is an unavoidable issue in pose estimation using 2D markerless motion-capture models. Previous studies have shown that knee joint measurements obtained from MediaPipe Pose under a frontal-plane view differ from those obtained using VICON [41]. Takeda et al. verified the feasibility of OpenPose measurement in a single anatomical plane [17], and differences in camera view have also been shown to influence OpenPose quantification results [42]. These findings, together with the present results, indicate that the quantification accuracy of 2D markerless motion capture is strongly influenced by changes in the joint movement plane.

When analyzing absolute joint angles, we simplified the plane of joint motion at each time point across the entire movement cycle as the plane formed by the proximal and distal limb segments composing the joint. During movement, this plane continuously changes in space as the action progresses. We speculate that when the plane is close to being perpendicular to the camera view, the angles quantified by 2D markerless motion capture may be closer to the synthesized angles obtained from Xsens. We consider that the differences in quantification among joints under the same view, as well as changes in the agreement of the same joint across different views, mainly arise from the interaction between the movement plane and the camera-view angle.

The results showed that changes in camera view altered the quantification performance of previously better-performing joints. However, this did not appear as an overall improvement in waveform similarity or a slight decrease in RMSE for all joints, but rather as the emergence of newly better-performing joints. Under the 15° camera view, the actual movement planes of the bilateral knee joints and the left hip joint were closer to a more favorable projection condition, which improved the quantification performance of these joints. When the camera view changed from 15° to 35°, the quantified results of the left shoulder joint obtained by MediaPipe Pose and DWPose improved. The increased camera angle reduced trunk occlusion of the elbow joint during movement, thereby improving the identification accuracy of the left elbow keypoints. In addition, we speculate that the left shoulder joint spent more time close to a more favorable projection condition under the 35° camera view, and the combined effect of these two factors improved the quantified results for the left shoulder joint.

Under the same camera view, MediaPipe Pose and DWPose generally quantified the knee joint better than the ankle and hip joints. In particular, during the backswing phase, the left knee showed relatively high agreement in the discrete variables. This may be because temporal alignment of the discrete variables in this study was based on predefined kinematic events. At this time point, the flexion–extension movement pattern of the left knee was relatively clear, and the joint movement plane was more favorable for a 2D projection of the true angle. However, this result does not indicate that the left knee had high quantification accuracy throughout the entire movement cycle. The continuous-variable analysis showed that the waveform similarity of the left knee was at a moderate level and that RMSE remained high. One possible reason is that, in the middle phase of the movement, the left leg served as the support leg and was planted to the left side of the ball. The football may have affected the prediction accuracy of the left ankle keypoints to some extent, thereby influencing the knee joint. In the middle-to-late phase of the movement, increased overlap and occlusion between the swinging right leg and the left leg may also have reduced the quantification stability of the left knee angle. The SPM and SnPM analyses showed that the early phase of the movement cycle had a greater influence on the right knee joint. In the early phase, the difference between the right knee movement plane and a more favorable projection condition was more pronounced than in the middle-to-late phase, resulting in significant differences in the early phase of the right knee movement.

### 4.3. Model-Level Analysis

MediaPipe Pose, DWPose, and YOLO-Pose differed in their quantification accuracy for the 30 m long-pass movement. MediaPipe Pose and DWPose showed better quantification performance for multiple joints, whereas YOLO-Pose generally performed worse than DWPose and MediaPipe Pose. The highest CMD value of YOLO-Pose was only 0.54 ± 0.25, and no clearly better-performing joint was observed. Although its RMSE values were similar to those of DWPose and MediaPipe Pose, the accuracy of its quantified results was also poorer than that of the other two models (see Supplementary Materials). In terms of keypoint identification accuracy, YOLO-Pose more frequently showed identification deviations for the bilateral wrist and ankle keypoints even without occlusion. In contrast, MediaPipe Pose improves robustness through special processing involving affine-normalized cropping of consecutive movement-sequence images [32]. DWPose is based on two-stage distillation and dual-dataset training [43], which improves its generalization ability and makes its model outputs more balanced.

The results of both discrete and continuous variables showed that the three models had relatively large standard deviations in the quantified results for some joints. This may be related to the complexity of the target joint movement and the occlusion of the joint by other body segments during movement. The calculation of joint angles by 2D markerless motion-capture models relies on the 2D coordinates of adjacent body keypoints. When the target joint involves complex multiplanar movement or is affected by occlusion, the difficulty of keypoint identification increases, which may reduce the stability of the quantified angles to some extent.

### 4.4. Limitations

Common electronic triggers or optical triggers are often used as hardware synchronization devices in high-precision validation studies. This study did not use a common electronic trigger or optical trigger to achieve hardware synchronization between Xsens and the mobile devices. Instead, a temporal alignment method based on predefined kinematic events was used. Although the precision of temporal alignment was lower than that of hardware synchronization, its practicality and convenience should not be overlooked. Second, the mobile devices were not subjected to rigorous spatial calibration or lens-distortion correction. Therefore, the joint angles quantified by 2D markerless motion capture may have been affected by perspective error and lens distortion. The purpose of this study was to preliminarily evaluate the agreement between 2D markerless motion-capture models combined with mobile devices and a field-deployable inertial motion-capture reference system, as well as their feasibility for rapid screening of movement patterns and analysis of movement trends under real football-field conditions, rather than to perform high-precision three-dimensional reconstruction. The absence of a complex calibration procedure is also consistent with the convenience and practicality required for field-based use.

This study used a fixed 30-frame analysis window to analyze the core phase of the long-pass movement. This approach cannot completely eliminate differences in movement rhythm among participants or the natural variability that occurs across repeated long-pass trials within the same participant.

### 4.5. Recommendations and Future Perspectives

Regarding the current application of 2D markerless motion-capture models in football-specific technical movement analysis, researchers should first optimize the camera view according to the overall motion pattern of the target technical movement and the key joints of interest before formal data collection and measurement. In the 15° condition, MediaPipe Pose and DWPose may be used for the rapid screening of motion patterns in the bilateral knee joints and left hip joint during the 30 m long pass, or for the preliminary identification of approximate phases of technical movement abnormalities, thereby providing clues for subsequent, more precise biomechanical analysis. However, the results of this study are not sufficient to support the use of MediaPipe Pose and DWPose for the high-precision quantification of joint angles.

Given that the movement planes of different joints do not change in the same way throughout the movement, when a study involves the simultaneous measurement of multiple joints, multiple camera views are recommended according to the general movement trends of different joints. At the same time, potential phase shifts during data integration should be considered. In addition, when MediaPipe Pose and DWPose are used for rapid assessment of movement trends, it is recommended to appropriately increase the number of valid trials and to pay attention to data screening and preprocessing, so as to improve the stability and interpretability of the results.

The recently proposed lightweight 2D pose-estimation network architecture LSDNet [44] emphasizes maintaining pose-estimation performance while reducing model complexity. If pretrained model weights such as those from MediaPipe Pose or DWPose are combined with transfer learning or secondary training to adapt to football-specific measurement scenarios, it may be possible to develop a lightweight markerless motion-capture model that is more suitable for real field environments while maintaining or even improving accuracy as much as possible. Meanwhile, specialized training of models for football-specific technical movement scenarios and the sharing of related football datasets may further improve the accuracy of keypoint identification under complex movement conditions and reduce the cost of data acquisition and annotation. Overall, 2D markerless motion capture has a relatively clear development prospect in the analysis of football-specific technical movements.

## 5. Conclusions

Based on a preliminary validation of agreement in the 30 m inside-foot long-pass scenario, none of the three 2D markerless motion-capture models showed stable high agreement with Xsens for either discrete or continuous variables. Therefore, current 2D markerless motion capture combined with mobile devices cannot yet be used for high-precision joint-angle measurement or precise clinical analysis of high-speed dynamic football-specific movements in real field settings. However, under the 15° camera view, the continuous-variable quantified results of MediaPipe Pose and DWPose for the bilateral knee joints and left hip joint reached a moderate level or above. This suggests that researchers, coaches, and players may selectively use these models for the rapid screening of movement patterns and analysis of movement trends in football-specific technical movements, based on the standardized operating procedure of this study and with clearly defined error boundaries. In contrast, YOLO-Pose did not show application performance comparable to that of MediaPipe Pose and DWPose, and its applicability in football-specific technical movement analysis requires further verification.

## Figures and Tables

**Table 1:** Agreement analysis of joint kinematic variables derived from single-frame MediaPipe Pose data.

		Camera View 1				Camera View 2			
Kinematic Phase	Joint	ICC (2,1)	95% CI	Bias	95% LoA	ICC (2,1)	95% CI	Bias	95% LoA
Backswing	Right Ankle	−0.07	(−0.12, −0.03)	30.94 *	(−11.91, 73.78)	−0.12	(−0.16, −0.08)	29.72 *	(−13.69, 73.12)
	Right Shoulder	0.15	(0.07, 0.24)	−16.16 *	(−43.37, 11.04)	0.46	(0.36, 0.56)	−9.40 *	(−28.79, 9.98)
	Right Knee	0.10	(0.04, 0.17)	24.87 *	(−7.78, 57.51)	−0.03	(−0.10, 0.05)	24.96 *	(−25.46, 75.37)
	Right Elbow	0.28	(0.23, 0.33)	−24.79 *	(−98.76, 49.18)	0.27	(0.22, 0.32)	−10.48	(−103.41, 82.44)
	Right Hip	0.25	(0.21, 0.29)	−9.90 *	(−21.90, 2.09)	0.09	(0.03, 0.15)	−10.07 *	(−22.13, 1.99)
	Left Ankle	−0.01	(−0.02, 0.00)	50.00 *	(21.56, 78.43)	0.03	(0.01, 0.05)	33.08 *	(9.99, 56.18)
	Left Shoulder	0.42	(0.24, 0.60)	−7.15 *	(−30.09, 15.78)	0.42	(0.28, 0.55)	0.76	(−22.47, 23.98)
	Left Knee	0.83	(0.75, 0.88)	−1.44 *	(−9.44, 6.56)	0.79	(0.71, 0.85)	−0.86 *	(−11.19, 9.47)
	Left Elbow	0.66	(0.51, 0.79)	−12.93 *	(−37.62, 11.75)	0.76	(0.67, 0.82)	6.67 *	(−27.02, 40.37)
	Left Hip	0.40	(0.19, 0.63)	1.69 *	(−17.26, 20.65)	0.31	(0.25, 0.36)	11.98 *	(−2.78, 26.74)
Follow-through	Right Ankle	0.11	(0.04, 0.18)	26.64 *	(−1.38, 54.65)	0.08	(0.02, 0.13)	35.05 *	(1.91, 68.19)
	Right Shoulder	0.41	(0.28, 0.50)	3.66 *	(−25.54, 32.85)	0.43	(0.29, 0.53)	7.54 *	(−17.88, 32.95)
	Right Knee	0.49	(0.38, 0.60)	4.33 *	(−6.80, 15.46)	0.50	(0.37, 0.62)	4.91 *	(−5.81, 15.62)
	Right Elbow	0.56	(0.50, 0.64)	−8.41	(−62.21, 45.39)	0.44	(0.30, 0.57)	9.17 *	(−16.46, 34.80)
	Right Hip	0.36	(0.26, 0.45)	4.29 *	(−22.61, 31.19)	0.02	(−0.03, 0.08)	19.25 *	(−8.13, 46.63)
	Left Ankle	0.10	(0.05, 0.16)	20.57 *	(−33.85, 74.99)	0.17	(0.06, 0.28)	19.98 *	(−38.94, 78.90)
	Left Shoulder	−0.02	(−0.39, 0.90)	2.80	(−22.19, 27.80)	0.61	(0.50, 0.70)	8.84 *	(−9.56, 27.24)
	Left Knee	0.75	(0.65, 0.84)	1.46 *	(−14.98, 17.90)	0.48	(0.36, 0.61)	7.58 *	(−9.50, 24.65)
	Left Elbow	0.76	(0.63, 0.87)	−2.07	(−21.86, 17.71)	0.24	(0.12, 0.36)	20.35 *	(−21.55, 62.25)
	Left Hip	0.39	(0.29, 0.49)	−4.97 *	(−22.57, 12.63)	0.41	(0.27, 0.55)	3.44 *	(−14.53, 21.42)

Note: Camera View 1 indicates that the camera was positioned 15° to the right-front of the participant, whereas Camera View 2 indicates that the camera was positioned 35° to the right-front of the participant. 95% CI represents the 95% confidence interval associated with ICC (2,1). * indicates a significant systematic bias between the two measurement methods (*p* < 0.05).

**Table 2:** Agreement analysis of joint kinematic variables derived from single-frame DWPose data.

		Camera View 1				Camera View 2			
Kinematic Phase	Joint	ICC (2,1)	95% CI	Bias	95% LoA	ICC (2,1)	95% CI	Bias	95% LoA
Backswing	Right Ankle	−0.04	(−0.09, 0.00)	23.02 *	(−10.30, 56.33)	−0.31	(−0.38, −0.23)	9.19 *	(−45.90, 64.27)
	Right Shoulder	0.12	(0.03, 0.22)	−13.06 *	(−41.52, 15.41)	0.51	(0.40, 0.62)	−7.01 *	(−26.96, 12.94)
	Right Knee	0.13	(0.04, 0.22)	17.19 *	(−14.56, 48.94)	0.08	(−0.02, 0.20)	14.08 *	(−40.75, 68.90)
	Right Elbow	0.29	(0.24, 0.34)	−25.66 *	(−97.31, 45.98)	0.27	(0.21, 0.33)	−13.70	(−105.73, 78.33)
	Right Hip	0.34	(0.28, 0.39)	−7.95 *	(−18.11, 2.21)	0.25	(0.19, 0.32)	−7.20 *	(−17.26, 2.86)
	Left Ankle	0.01	(−0.01, 0.03)	29.34 *	(7.77, 50.91)	0.05	(0.03, 0.09)	21.09 *	(4.72, 37.47)
	Left Shoulder	0.15	(0.08, 0.22)	−28.80 *	(−63.38, 5.77)	0.57	(0.48, 0.64)	−11.90 *	(−44.60, 20.79)
	Left Knee	0.81	(0.74, 0.86)	2.40 *	(−5.19, 10.00)	0.78	(0.69, 0.85)	2.75 *	(−5.57, 11.08)
	Left Elbow	0.43	(0.25, 0.59)	−14.07 *	(−72.92, 44.78)	0.67	(0.57, 0.74)	5.03 *	(−35.82, 45.88)
	Left Hip	0.28	(0.14, 0.42)	8.11 *	(−10.34, 26.55)	0.18	(0.15, 0.21)	17.35 *	(4.31, 30.40)
Follow-through	Right Ankle	0.18	(0.08, 0.27)	18.43 *	(−8.40, 45.26)	0.20	(0.11, 0.29)	18.53 *	(−8.35, 45.41)
	Right Shoulder	0.39	(0.28, 0.49)	7.22 *	(−22.64, 37.08)	0.49	(0.40, 0.58)	9.19 *	(−16.16, 34.55)
	Right Knee	0.51	(0.39, 0.62)	4.65 *	(−5.56, 14.87)	0.31	(0.17, 0.43)	5.80 *	(−6.25, 17.86)
	Right Elbow	0.60	(0.54, 0.67)	−9.14 *	(−57.15, 38.88)	0.52	(0.41, 0.62)	8.80 *	(−24.70, 42.29)
	Right Hip	0.35	(0.25, 0.44)	4.96 *	(−20.80, 30.72)	0.13	(0.07, 0.20)	17.82 *	(−7.77, 43.41)
	Left Ankle	0.20	(0.11, 0.28)	6.22 *	(−46.32, 58.76)	0.24	(0.14, 0.33)	7.14 *	(−44.66, 58.93)
	Left Shoulder	0.66	(0.57, 0.75)	−1.57 *	(−28.77, 25.64)	0.80	(0.76, 0.84)	1.76	(−19.30, 22.82)
	Left Knee	0.68	(0.61, 0.75)	6.07 *	(−9.72, 21.86)	0.50	(0.40, 0.59)	9.86 *	(−3.22, 22.94)
	Left Elbow	0.25	(0.09, 0.40)	8.56 *	(−34.38, 51.51)	0.30	(0.18, 0.42)	18.88 *	(−20.16, 57.91)
	Left Hip	0.49	(0.37, 0.59)	1.39	(−15.47, 18.26)	0.25	(0.15, 0.37)	8.76 *	(−9.89, 27.42)

Note: Camera View 1 indicates that the camera was positioned 15° to the right-front of the participant, whereas Camera View 2 indicates that the camera was positioned 35° to the right-front of the participant. 95% CI represents the 95% confidence interval associated with ICC (2,1). * indicates a significant systematic bias between the two measurement methods (*p* < 0.05).

## Data Availability

All experimental data and codes have been deposited in the Zenodo repository: https://doi.org/10.5281/zenodo.19591213.

## Supplementary Materials

The following supporting information can be downloaded at: https://www.mdpi.com/article/10.3390/s26123654/s1, Supplementary Material S1: Bland–Altman plots for discrete kinematic agreement between markerless motion-capture models and Xsens; Supplementary Material S2: Additional agreement analyses and sample-size information for markerless motion-capture models; Supplementary Material S3: Additional SPM and SnPM comparisons of joint-angle time series.
