# Supplementary material for: Comparative Analysis of Markerless Motion-Capture Models for Assessing Football Kinematics During 30 m Long-Pass Tasks
Source: Sensors (Basel). 2026 Jun 8;26(12):3654. doi: 10.3390/s26123654 (PMC13306328; doi:10.3390/s26123654)
Supplement: Supplementary file 1 [file sensors-26-03654-s001.zip › Supplementary Material S1.pdf]

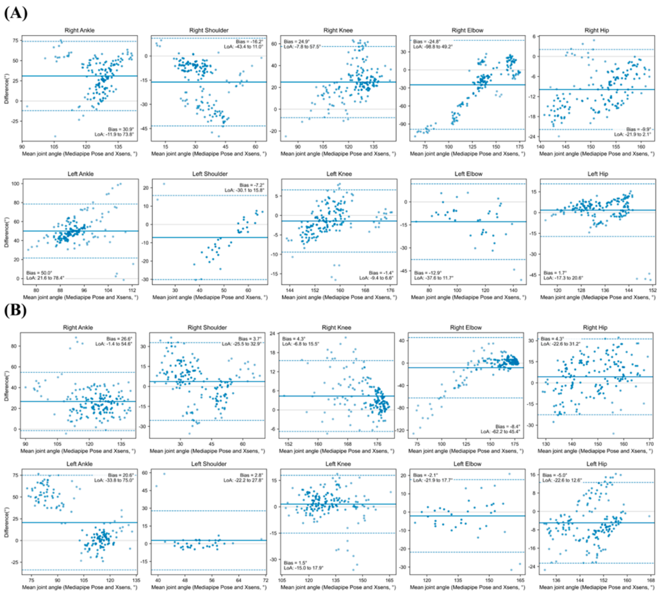

Figure S1. Bland–Altman analysis of discrete kinematic variables between Mediapipe Pose and Xsens at a 15° camera viewing angle. Note:**(A)** Backswing phase; **(B)** Follow-through phase. Solid lines indicate mean bias, and dashed lines represent 95% limits of agreement (±1.96 SD). Points represent individual trials.

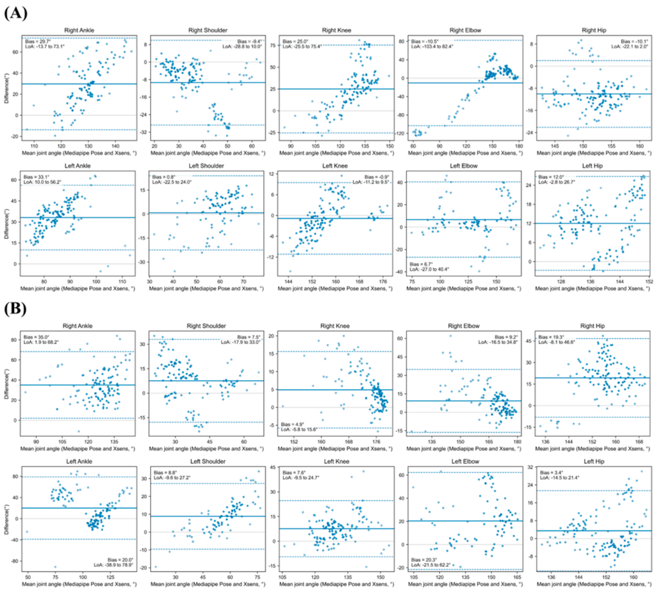

Figure S2. Bland–Altman analysis of discrete kinematic variables between Mediapipe Pose and Xsens at a 35° camera viewing angle. Note:**(A)** Backswing phase; **(B)** Follow-through phase. Solid lines indicate mean bias, and dashed lines represent 95% limits of agreement (±1.96 SD). Points represent individual trials.

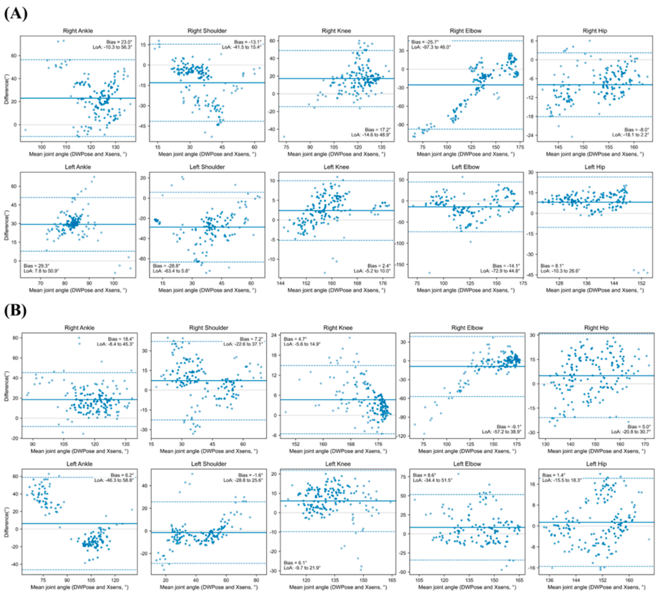

Figure S3. Bland–Altman analysis of discrete kinematic variables between DWPose and Xsens at a 15° camera viewing angle. Note:**(A)** Backswing phase; **(B)** Follow-through phase. Solid lines indicate mean bias, and dashed lines represent 95% limits of agreement (±1.96 SD). Points represent individual trials.

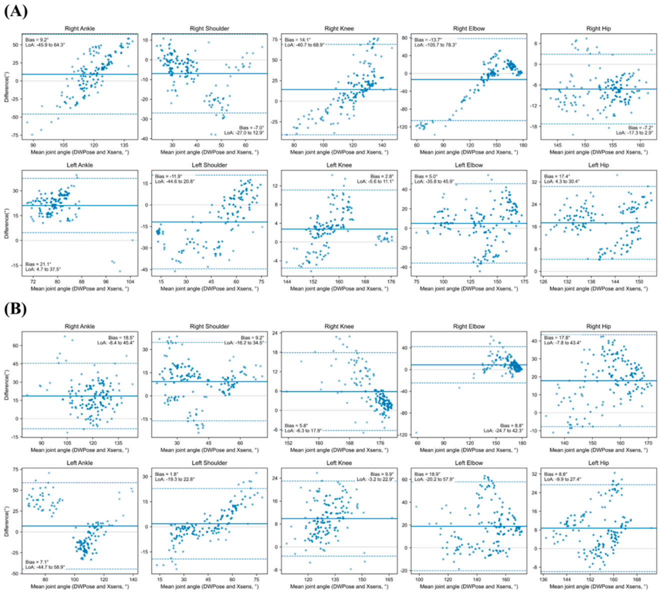

Figure S4. Bland–Altman analysis of discrete kinematic variables between DWPose and Xsens at a 35° camera viewing angle. Note:**(A)** Backswing phase; **(B)** Follow-through phase. Solid lines indicate mean bias, and dashed lines represent 95% limits of agreement (±1.96 SD). Points represent individual trials.

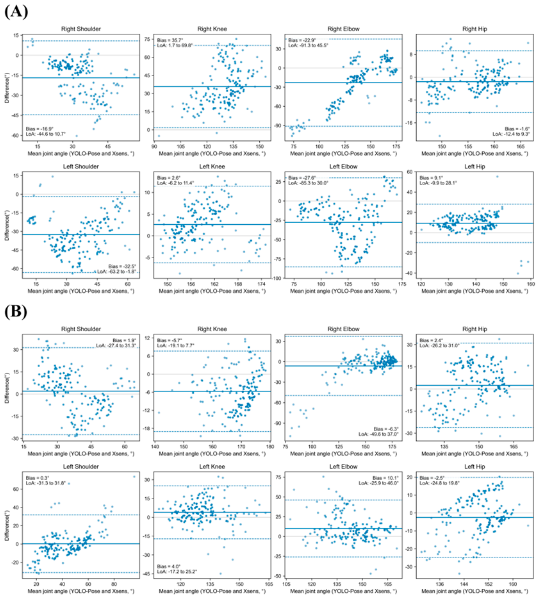

Figure S5. Bland–Altman analysis of discrete kinematic variables between YOLO-Pose and Xsens at a 15° camera viewing angle. Note:**(A)** Backswing phase; **(B)** Follow-through phase. Solid lines indicate mean bias, and dashed lines represent 95% limits of agreement (±1.96 SD). Points represent individual trials.
