# Supplementary material for: Comparative Analysis of Markerless Motion-Capture Models for Assessing Football Kinematics During 30 m Long-Pass Tasks
Source: Sensors (Basel). 2026 Jun 8;26(12):3654. doi: 10.3390/s26123654 (PMC13306328; doi:10.3390/s26123654)
Supplement: Supplementary file 1 [file sensors-26-03654-s001.zip › Supplementary Material S2.pdf]

Tabel S1. Agreement analysis of joint kinematic variables derived from single-frame YOLO-Pose data

		Camera View 1				Camera View 2			
**Kinematic phase**	**Angle**	**ICC (2,1)**	**95% CI**	**Bias**	**95% LoA**	**ICC (2,1)**	**95% CI**	**Bias**	**95% LoA**
Backswing	Right Shoulder	0.10	(0.03, 0.17)	-16.94*	(-44.60, 10.73)	0.40	(0.29, 0.49)	-9.89*	(-33.15, 13.37)
	Right Knee	0.05	(0.02, 0.09)	35.73*	(1.70, 69.75)	0.03	(-0.00, 0.08)	40.34*	(-3.83, 84.52)
	Right Elbow	0.31	(0.26, 0.36)	-22.90*	(-91.26, 45.47)	0.30	(0.25, 0.35)	-17.12*	(-98.71, 64.47)
	Right Hip	0.53	(0.43, 0.61)	-1.56*	(-12.38, 9.25)	0.44	(0.32, 0.54)	-1.03*	(-12.61, 10.55)
	Left Shoulder	0.14	(0.10, 0.19)	-32.52*	(-63.23, -1.82)	0.44	(0.37, 0.52)	-13.64*	(-52.74, 25.46)
	Left Knee	0.72	(0.64, 0.79)	2.60*	(-6.22, 11.42)	0.61	(0.52, 0.69)	2.23*	(-9.91, 14.38)
	Left Elbow	0.28	(0.19, 0.38)	-27.64*	(-85.27, 29.99)	0.31	(0.16, 0.45)	-7.81	(-66.79, 51.17)
	Left Hip	0.28	(0.17, 0.38)	9.08*	(-9.90, 28.06)	0.13	(0.07, 0.19)	14.68*	(-3.63, 32.98)
Follow-through	Right Shoulder	0.37	(0.24, 0.48)	1.94	(-27.44, 31.32)	0.40	(0.28, 0.50)	5.83*	(-21.75, 33.41)
	Right Knee	0.50	(0.39, 0.59)	-5.71*	(-19.08, 7.66)	0.21	(0.08, 0.33)	-4.96*	(-25.74, 15.81)
	Right Elbow	0.63	(0.56, 0.72)	-6.30*	(-49.57, 36.97)	0.49	(0.43, 0.57)	2.58*	(-47.92, 53.08)
	Right Hip	0.31	(0.21, 0.41)	2.41*	(-26.22, 31.04)	0.05	(-0.01, 0.12)	16.01*	(-17.09, 49.10)
	Left Shoulder	0.60	(0.50, 0.69)	0.28	(-31.25, 31.81)	0.68	(0.58, 0.76)	5.54*	(-20.97, 32.05)
	Left Knee	0.58	(0.45, 0.70)	3.97*	(-17.22, 25.16)	0.42	(0.27, 0.55)	7.88*	(-15.44, 31.20)
	Left Elbow	0.32	(0.19, 0.45)	10.05*	(-25.93, 46.04)	0.17	(0.07, 0.28)	17.58*	(-25.24, 60.39)
	Left Hip	0.24	(0.11, 0.35)	-2.47*	(-24.77, 19.83)	0.43	(0.33, 0.51)	6.02*	(-9.57, 21.61)

Note:Camera View 1 indicates that the camera was positioned 15° to the right-front of the participant, whereas Camera View 2 indicates that the camera was positioned 35° to the right-front of the participant. 95% CI represents the 95% confidence interval associated with ICC (2,1); * indicates a significant systematic bias between the two measurement methods ($p < 0.05$).

Table S2. Sample Size of Discrete Motion Variables in DWPose

	Backswing				
	**Right Ankle**	**Right Shoulder**	**Right Knee**	**Right Elbow**	**Right Hip**
Camera	153	159	159	159	159
view 1	**Left Ankle**	**Left Shoulder**	**Left Knee**	**Left Elbow**	**Left Hip**
	137	134	159	127	159
	**Right Ankle**	**Right Shoulder**	**Right Knee**	**Right Elbow**	**Right Hip**
Camera	140	143	143	142	143
view 2	**Left Ankle**	**Left Shoulder**	**Left Knee**	**Left Elbow**	**Left Hip**
	124	130	132	141	132
	Follow-through				
	**Right Ankle**	**Right Shoulder**	**Right Knee**	**Right Elbow**	**Right Hip**
Camera	157	163	163	163	163
view 1	**Left Ankle**	**Left Shoulder**	**Left Knee**	**Left Elbow**	**Left Hip**
	141	134	159	128	159
	**Right Ankle**	**Right Shoulder**	**Right Knee**	**Right Elbow**	**Right Hip**
Camera	140	143	143	142	143
view 2	**Left Ankle**	**Left Shoulder**	**Left Knee**	**Left Elbow**	**Left Hip**
	124	130	132	141	132

Note: Camera View 1 is at a 15° position, and Camera View 2 is at a 35° position.

Table S3. Sample Size of Discrete Motion Variables in Mediapipe Pose

	Backswing				
	**Right Ankle**	**Right Shoulder**	**Right Knee**	**Right Elbow**	**Right Hip**
Camera	155	158	158	158	158
view 1	**Left Ankle**	**Left Shoulder**	**Left Knee**	**Left Elbow**	**Left Hip**
	158	37	158	37	158
	**Right Ankle**	**Right Shoulder**	**Right Knee**	**Right Elbow**	**Right Hip**
Camera	113	139	123	137	139
view 2	**Left Ankle**	**Left Shoulder**	**Left Knee**	**Left Elbow**	**Left Hip**
	139	95	139	94	139
	Follow-through				
	**Right Ankle**	**Right Shoulder**	**Right Knee**	**Right Elbow**	**Right Hip**
Camera	160	163	163	163	163
view 1	**Left Ankle**	**Left Shoulder**	**Left Knee**	**Left Elbow**	**Left Hip**
	158	37	158	37	158

	Right Ankle	Right Shoulder	Right Knee	Right Elbow	Right Hip
Camer	117	127	127	126	127
view 2	**Left Ankle**	**Left Shoulder**	**Left Knee**	**Left Elbow**	**Left Hip**
	123	85	123	84	126

Note: Camera View 1 is at a 15° position, and Camera View 2 is at a 35° position.

Table S4. Sample Size of Discrete Motion Variables in YOLO-Pose

	Backswing			
	**Right Shoulder**	**Right Knee**	**Right Elbow**	**Right Hip**
Camera	163	163	163	163
view 1	**Left Shoulder**	**Left Knee**	**Left Elbow**	**Left Hip**
	163	163	163	163
	**Right Shoulder**	**Right Knee**	**Right Elbow**	**Right Hip**
Camera	162	162	162	162
view 2	**Left Shoulder**	**Left Knee**	**Left Elbow**	**Left Hip**
	162	162	162	162
	Follow-through			
	**Right Shoulder**	**Right Knee**	**Right Elbow**	**Right Hip**
Camera	163	163	163	163
view 1	**Left Shoulder**	**Left Knee**	**Left Elbow**	**Left Hip**
	163	163	163	163
	**Right Shoulder**	**Right Knee**	**Right Elbow**	**Right Hip**
Camera	162	162	162	162
view 2	**Left Shoulder**	**Left Knee**	**Left Elbow**	**Left Hip**
	162	162	162	162

Table S5. Sample Size Used for SPM and SnPM Analyses

Joint	DWPose		Mediapipe Pose		YOLO-Pose	
	15°	35°	15°	35°	15°	35°
Right Ankle	12	10	12	10	12	10
Right Shoulder	12	10	12	10	12	10
Right Knee	12	10	12	10	12	10
Right Elbow	12	10	12	10	12	10
Right Hip	12	10	12	10	12	10
Left Ankle	12	10	12	10	12	10
Left Shoulder	12	10	4	9	12	10

Left Knee	12	10	12	10	12	10
Left Elbow	12	10	4	10	12	10
Left Hip	12	10	12	10	12	10

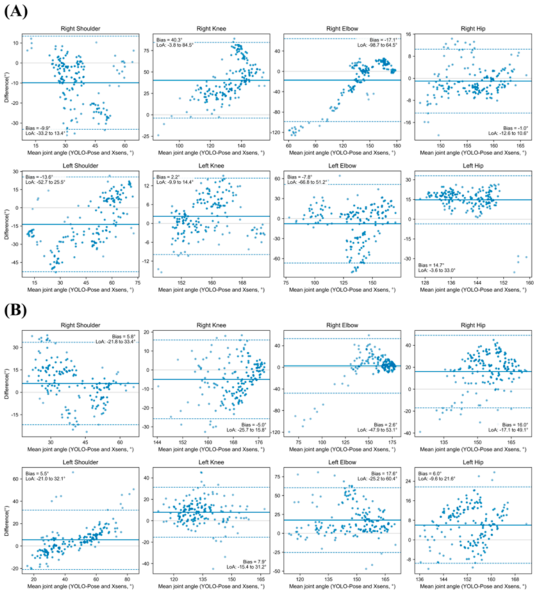

Figure S6. Bland–Altman analysis of discrete kinematic variables between DWPose and Xsens at a 15° camera viewing angle. Note:**(A)** Backswing phase; **(B)** Follow-through phase. Solid lines indicate mean bias, and dashed lines represent 95% limits of agreement (±1.96 SD). Points represent individual trials.

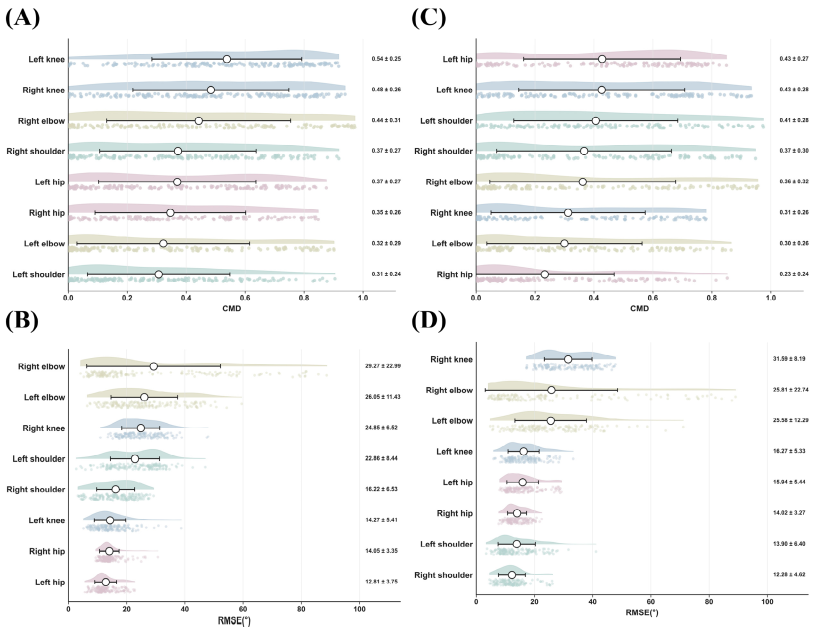

Figure S7. Comparison of joint kinematic agreement between YOLO-Pose and Xsens at two camera viewing angles (15° and 35°).Note:(**A**, **C**) CMD values representing waveform similarity.(**B**, **D**) RMSE values representing absolute angular differences. Panels (**A**, **B**) correspond to 15°, and (**C**, **D**) correspond to 35°. Data are presented as individual trials with mean ± SD.

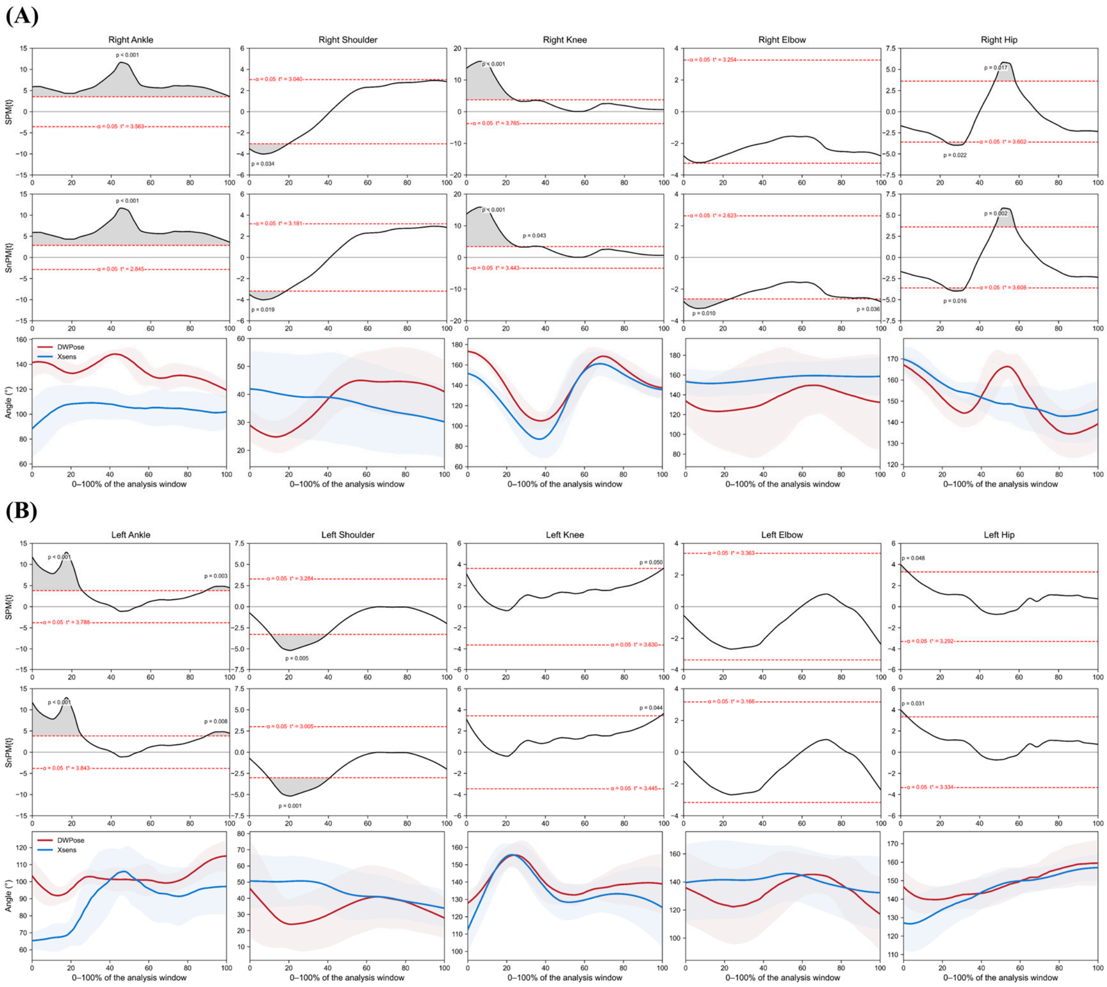

Figure S8. SPM and SnPM comparisons of bilateral joint angle time series between DWPose and Xsens over the fixed analysis window (0–100%) at a 15° camera angle.Note:(**A**) Right-side joints, including the right ankle, right shoulder, right knee, right elbow, and right hip.(**B**) Left-side joints, including the left ankle, left shoulder, left knee, left elbow, and left hip.Each column represents one joint. For each joint, the upper row presents the SPM{t} result, the middle row presents the SnPM{t} result, and the lower row presents the mean joint-angle trajectories quantified by DWPose and Xsens. The black solid curve represents the test statistic across the fixed analysis window. Red dashed lines indicate the critical thresholds of the test statistic at $\alpha = 0.05$, denoted as t* in the figure. Grey shaded regions represent supra-threshold clusters, indicating time regions with significant differences between DWPose and Xsens, with corresponding p values annotated in the figure. In the lower row, the red and blue curves represent DWPose and Xsens, respectively, and the shaded regions indicate variability across participants. The x-axis represents the fixed analysis window (%).
