# Supplementary material for: Comparative Analysis of Markerless Motion-Capture Models for Assessing Football Kinematics During 30 m Long-Pass Tasks
Source: Sensors (Basel). 2026 Jun 8;26(12):3654. doi: 10.3390/s26123654 (PMC13306328; doi:10.3390/s26123654)
Supplement: Supplementary file 1 [file sensors-26-03654-s001.zip › Supplementary Material S3.pdf]

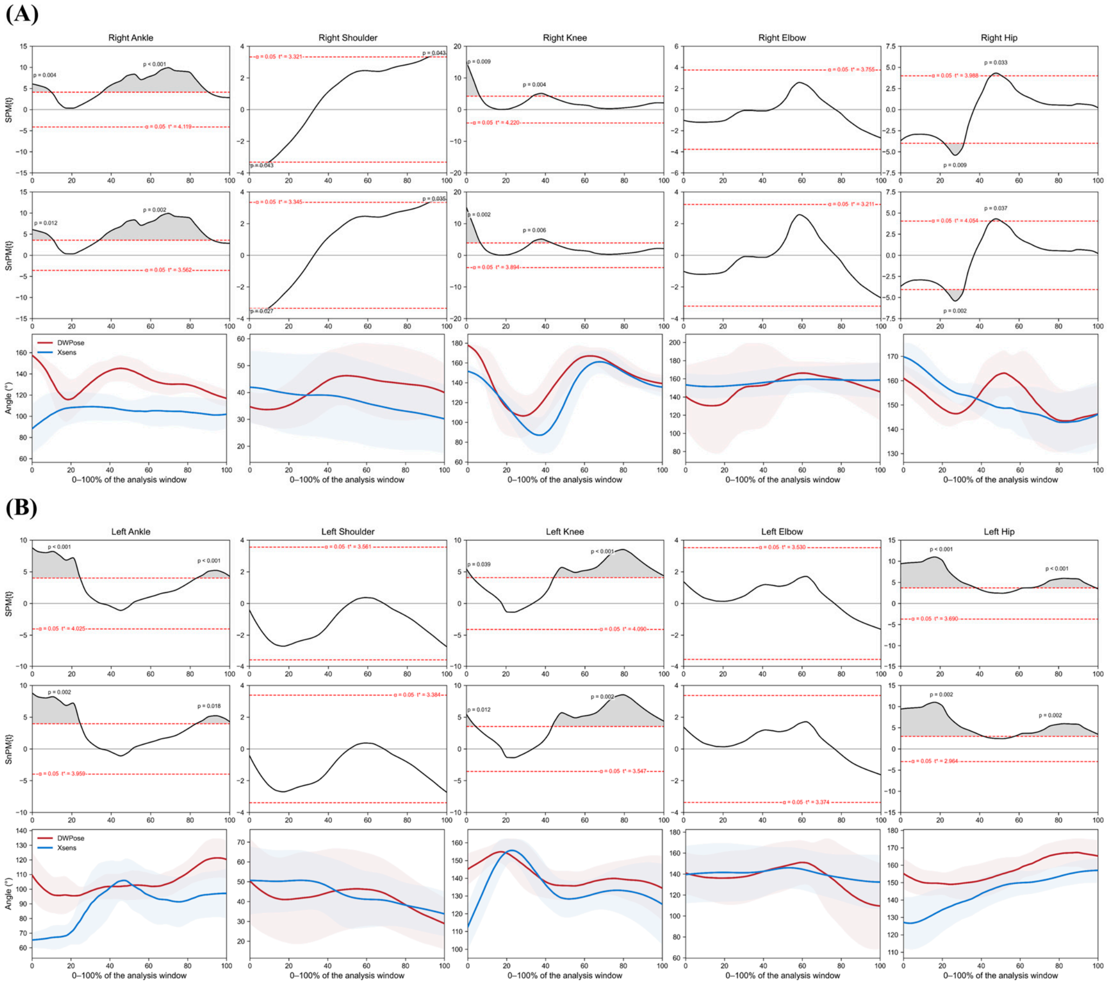

Figure S9. SPM and SnPM comparisons of bilateral joint angle time series between DWPose and Xsens over the fixed analysis window (0–100%) at a 35° camera angle.Note:(**A**) Right-side joints, including the right ankle, right shoulder, right knee, right elbow, and right hip.(**B**) Left-side joints, including the left ankle, left shoulder, left knee, left elbow, and left hip.Each column represents one joint. For each joint, the upper row presents the SPM{t} result, the middle row presents the SnPM{t} result, and the lower row presents the mean joint-angle trajectories quantified by DWPose and Xsens. The black solid curve represents the test statistic across the fixed analysis window. Red dashed lines indicate the significance thresholds at α = 0.05. Grey shaded regions represent supra-threshold clusters, indicating time regions with significant differences between DWPose and Xsens, with corresponding p values annotated in the figure. In the lower row, the red and blue curves represent DWPose and Xsens, respectively, and the shaded regions indicate variability across participants. The x-axis represents the fixed analysis window (%).

(A)

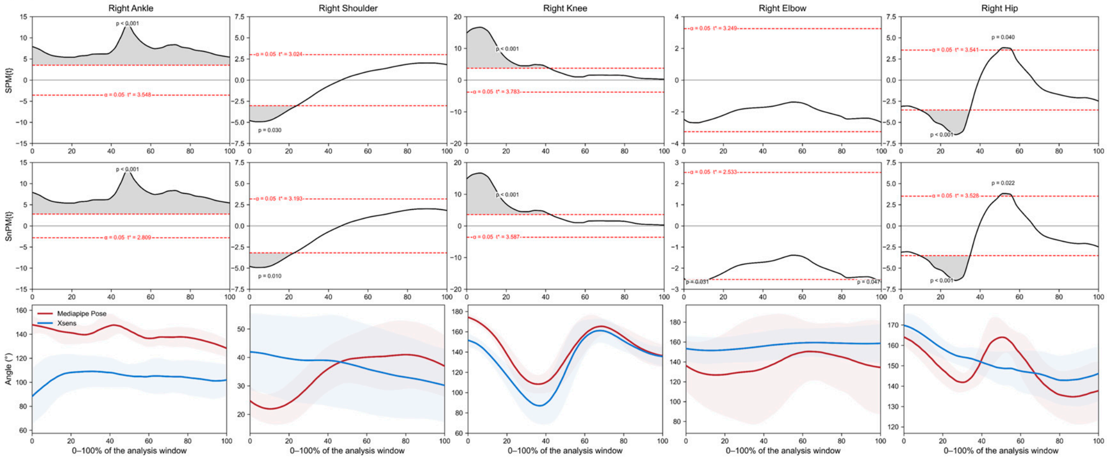

(B)

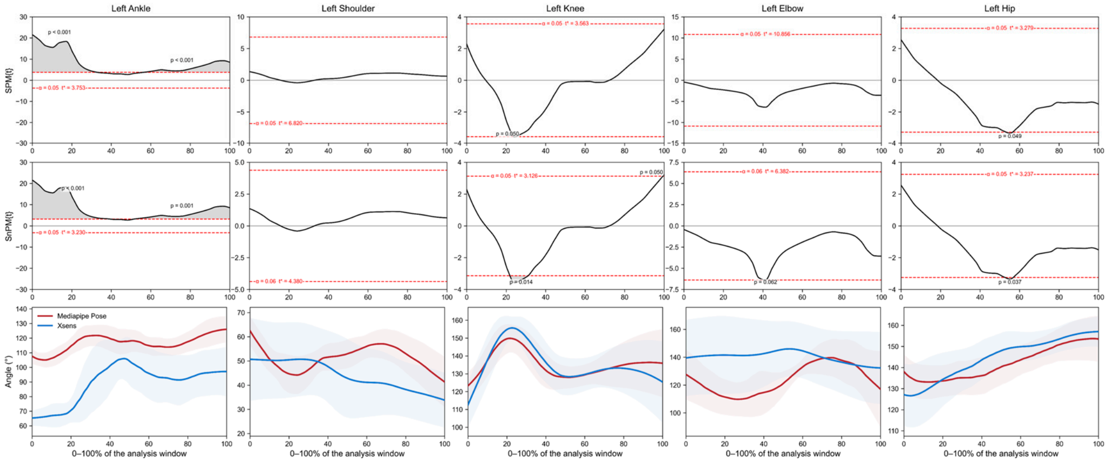

Figure S10. SPM and SnPM comparisons of bilateral joint angle time series between Mediapipe Pose and Xsens over the fixed analysis window (0–100%) at a 15° camera angle.Note:(**A**) Right-side joints, including the right ankle, right shoulder, right knee, right elbow, and right hip.(**B**) Left-side joints, including the left ankle, left shoulder, left knee, left elbow, and left hip.Each column represents one joint. For each joint, the upper row presents the SPM{t} result, the middle row presents the SnPM{t} result, and the lower row presents the mean joint-angle trajectories quantified by Mediapipe Pose and Xsens. The black solid curve represents the test statistic across the fixed analysis window. Red dashed lines indicate the critical thresholds of the test statistic at $\alpha = 0.05$, denoted as t* in the figure. Grey shaded regions represent supra-threshold clusters, indicating time regions with significant differences between Mediapipe Pose and Xsens, with corresponding p values annotated in the figure. In the lower row, the red and blue curves represent Mediapipe Pose and Xsens, respectively, and the shaded regions indicate variability across participants. The x-axis represents the fixed analysis window (%).

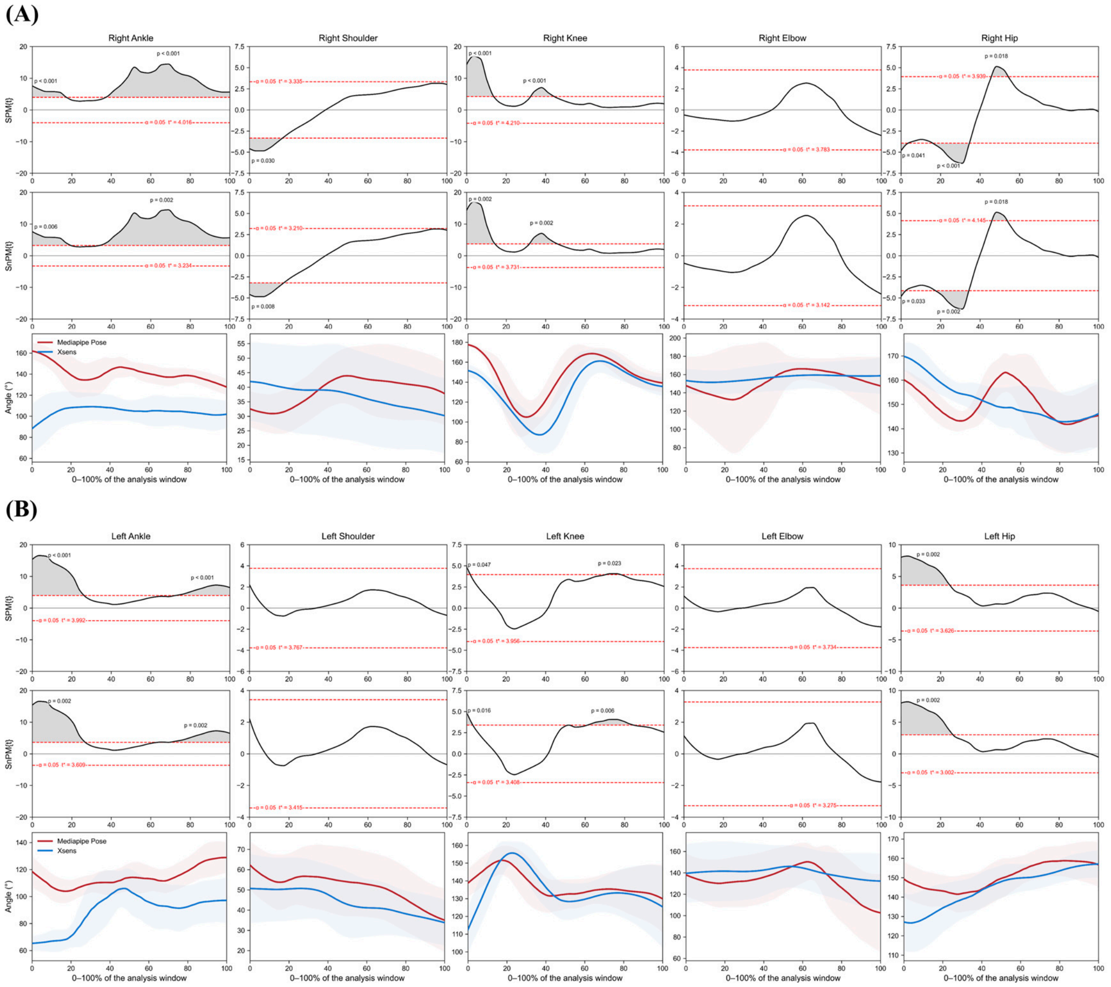

Figure S11. SPM and SnPM comparisons of bilateral joint angle time series between Mediapipe Pose and Xsens over the fixed analysis window (0–100%) at a 35° camera angle.Note:(**A**) Right-side joints, including the right ankle, right shoulder, right knee, right elbow, and right hip.(**B**) Left-side joints, including the left ankle, left shoulder, left knee, left elbow, and left hip.Each column represents one joint. For each joint, the upper row presents the SPM{t} result, the middle row presents the SnPM{t} result, and the lower row presents the mean joint-angle trajectories quantified by Mediapipe Pose and Xsens. The black solid curve represents the test statistic across the fixed analysis window. Red dashed lines indicate the critical thresholds of the test statistic at α = 0.05, denoted as t* in the figure. Grey shaded regions represent supra-threshold clusters, indicating time regions with significant differences between Mediapipe Pose and Xsens, with corresponding p values annotated in the figure. In the lower row, the red and blue curves represent Mediapipe Pose and Xsens, respectively, and the shaded regions indicate variability across participants. The x-axis represents the fixed analysis window (%).

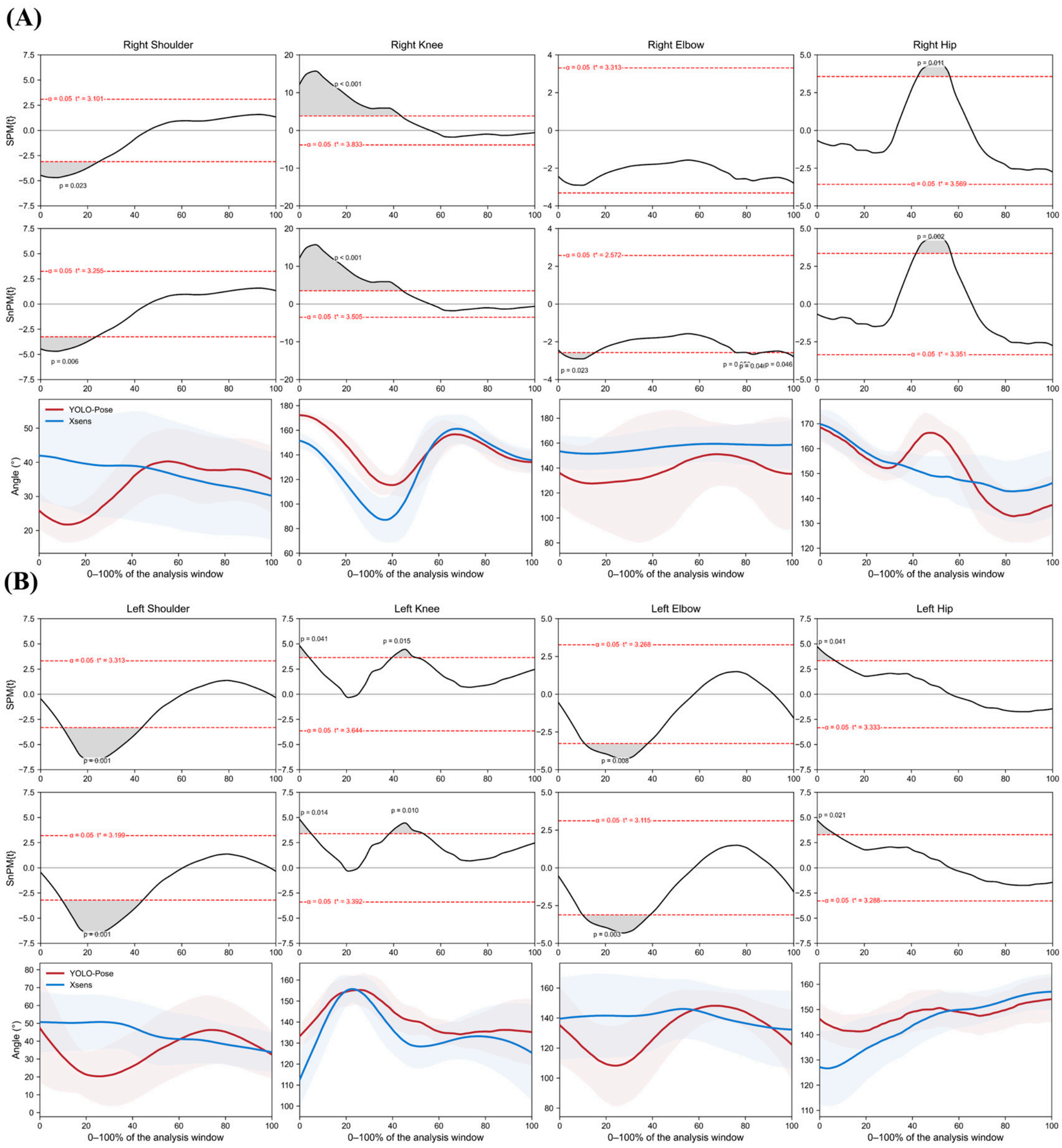

Figure S12. SPM and SnPM comparisons of bilateral joint angle time series between YOLO-Pose and Xsens over the fixed analysis window (0–100%) at a 15° camera angle.Note:(**A**) Right-side joints, including the right ankle, right shoulder, right knee, right elbow, and right hip.(**B**) Left-side joints, including the left ankle, left shoulder, left knee, left elbow, and left hip.Each column represents one joint. For each joint, the upper row presents the SPM{t} result, the middle row presents the SnPM{t} result, and the lower row presents the mean joint-angle trajectories quantified by YOLO-Pose and Xsens. The black solid curve represents the test statistic across the fixed analysis window. Red dashed lines indicate the critical thresholds of the test statistic at $\alpha = 0.05$, denoted as t* in the figure. Grey shaded regions represent supra-threshold clusters, indicating time regions with significant differences between YOLO-Pose and Xsens, with corresponding p values annotated in the figure. In the lower row, the red and blue curves represent YOLO-Pose and Xsens, respectively, and the shaded regions indicate variability across participants. The x-axis represents the fixed analysis window (%).

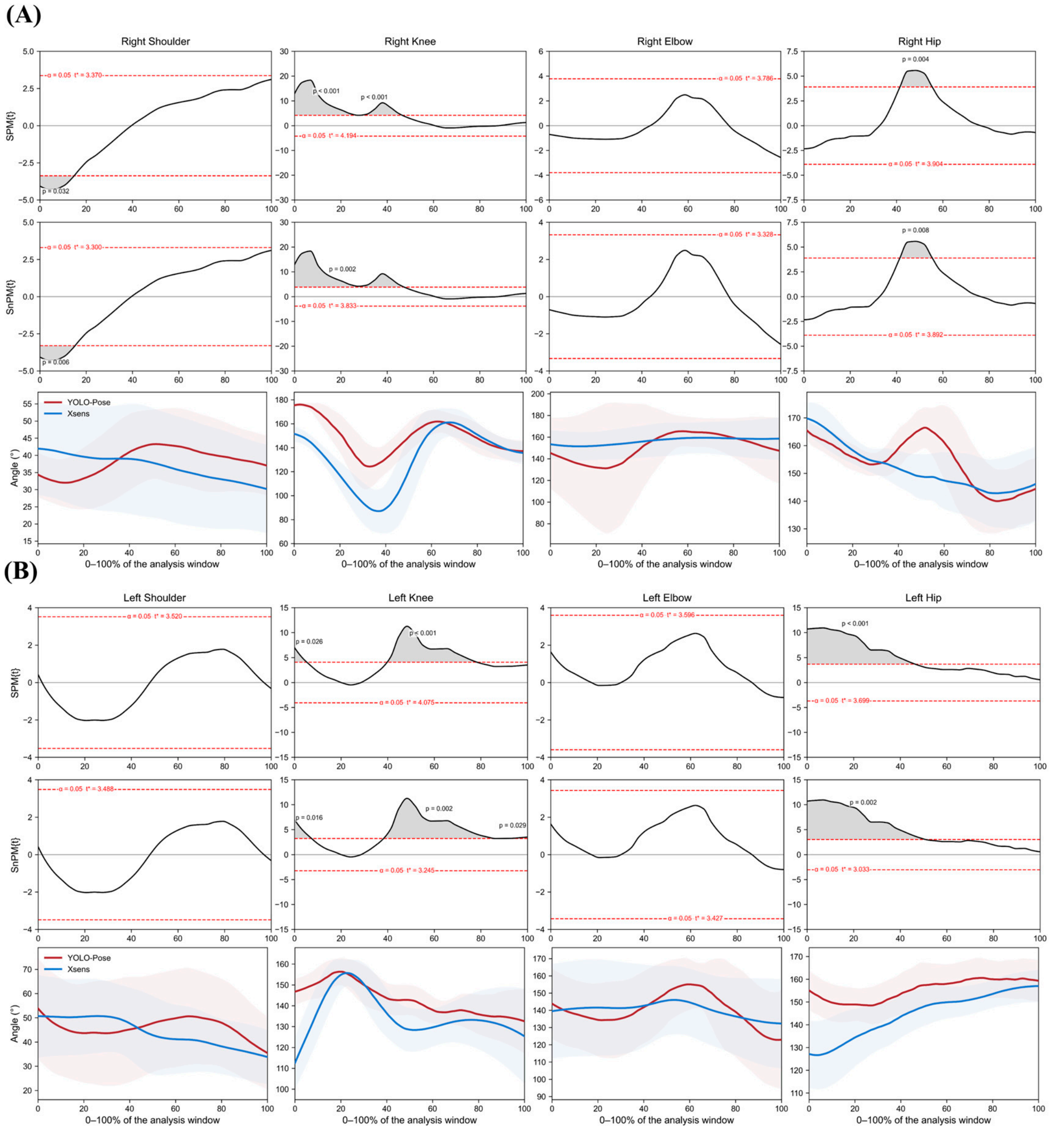

Figure S13. SPM and SnPM comparisons of bilateral joint angle time series between YOLO-Pose and Xsens over the fixed analysis window (0–100%) at a 15° camera angle.Note:(**A**) Right-side joints, including the right ankle, right shoulder, right knee, right elbow, and right hip.(**B**) Left-side joints, including the left ankle, left shoulder, left knee, left elbow, and left hip.Each column represents one joint. For each joint, the upper row presents the SPM{t} result, the middle row presents the SnPM{t} result, and the lower row presents the mean joint-angle trajectories quantified by YOLO-Pose and Xsens. The black solid curve represents the test statistic across the fixed analysis window. Red dashed lines indicate the critical thresholds of the test statistic at α = 0.05, denoted as t* in the figure. Grey shaded regions represent supra-threshold clusters, indicating time regions with significant differences between YOLO-Pose and Xsens, with corresponding p values annotated in the figure. In the lower row, the red and blue curves represent YOLO-Pose and Xsens, respectively, and the shaded regions indicate variability across participants. The x-axis represents the fixed analysis window (%).
